# Clinical characteristics of Epstein–Barr virus infection in the pediatric nervous system

**DOI:** 10.1186/s12879-020-05623-1

**Published:** 2020-11-25

**Authors:** Huan Cheng, Doudou Chen, Xiaoling Peng, Peng Wu, Li Jiang, Yue Hu

**Affiliations:** 1 Department of Neurology, Children’s Hospital of Chongqing Medical University, No.136 Zhongshan 2nd Road, Yu Zhong District, Chongqing, 400014 China; 2 Ministry of Education Key Laboratory of Child Development and Disorders, Chongqing, China; 3 National Clinical Research Center for Child Health and Disorders (Chongqing), Chongqing, China; 4 China International Science and Technology Cooperation base of Child Development and Critical Disorders, Chongqing, China; 5 Chongqing Key Laboratory of Pediatrics, Chongqing, China; 6 Division of Science and Technology, Beijing Normal University-Hongkong Baptist University United International College, Zhuhai, China

**Keywords:** EBV, Nervous system, Encephalitis, Hematological system, Demyelination

## Abstract

**Background:**

To investigate the clinical characteristics of Epstein–Barr virus (EBV) infection in the pediatric nervous system (NS).

**Methods:**

We retrospectively analyzed the clinical data and follow-up results of 89 children with neurological damage caused by EBV who were hospitalized in the children’s hospital of Chongqing Medical University from January 2008 to April 2019.

**Results:**

EBV infection of the NS can occur at any time of the year. The highest incidence was seen in the age group of 0–4 years. Fever is the main clinical feature (74/89, 83.1%). The main clinical types were encephalitis/meningoencephalitis (64/89, 71.9%), acute myelitis (2/89, 2.2%), acute disseminated encephalomyelitis (ADEM) (3/89, 3.4%), Guillain–Barré Syndrome (GBS) (15/89, 16.9%), neurological damage caused by EBV-hemophagocytic lymphohistiocytosis (EBV-HLH) (4/89, 4.5%), and NS-post-transplant lymphoproliferative disorder (NS-PTLD) (1/89, 1.1%). Anti-N-methyl-D-aspartate receptor encephalitis was found during the convalescence of EBV encephalitis. EBV encephalitis/meningitis showed no symptoms of tonsillitis, lymph node enlargement, skin rash, hepatosplenomegaly. Acute motor axonal neuropathy is the chief complication in GBS caused by EBV.

**Conclusion:**

There were significant differences in neurological complications caused by EBV. The prognosis of EBV infection in the NS is generally good. These illnesses are often self-limiting. A few cases may show residual sequelae.

## Background

Epstein–Barr virus (EBV) is a common lymphocytic human herpesvirus, formally belonging to the Gammaherpesvirinae subfamily. EBV infection in children is non-specific, mainly characterized by respiratory symptoms. The neurological complications of EBV infection are relatively rare, about 0.4–7.5% [1]. The pathogenesis of neurological diseases associated with EBV infection is not fully understood. Currently, three modes of pathogenesis are identified: (1) The virus directly invades the nervous system (NS): most children with EBV viral encephalitis have no symptoms of EBV infection outside of the NS, such as tonsillitis, enlarged lymph nodes, skin rash, and hepatosplenomegaly. EBV encephalitis in children is suggested to be a primary neurological infection [2]. The viral DNA in the cerebrospinal fluid (CSF) disappears when the neurological symptoms of the disease improve, especially before the decrease of leukocytes in the CSF, which proves that neurological diseases are caused by direct virus invasion. (2) Immune-mediated infection: compared with other herpesviruses, EBV can cause immune-mediated symptoms in the NS, which may be related to the age and immune status of the host. EBV may share a common antigen with neurological myelin oligodendrocyte glycoprotein [3], which makes the immune system produce autoimmune T lymphocytes and anti-neuronal antibodies to autoantigens [4]. (3) Reactivation of latent infection: when the ratio of EBV antibody titer in the CSF and serum is larger than the ratio of serum gamma globulin concentration in the CSF and serum, suggesting that the specific EBV antibody is produced in the sheath, and EBV infection in the NS is reactivated after primary infection. Reactivation of latent infection may be the main pathogenic mechanism of neurological disease, especially when the patient is in a state of immunosuppression [5].

About 25% children with EBV infection could test positive for CSF antibodies but without obvious neurological symptoms. Most EBV infections are not specific and only cause mild neurological symptoms. Therefore, the neurological damage caused by EBV infection can be underestimated in the clinic. This article analyzes the retrospective clinical data and follow-up results of 89 children with neurological damage caused by EBV infection and provides evidence for diagnosis and treatment of neurological damage caused by EBV virus infection.

## Methods

### Patient enrollment and diagnosis

We retrospectively analyzed the clinical characteristics, auxiliary examination results, treatment, and prognosis of 89 children with neurological damage caused by EBV in the Children’s Hospital of Chongqing Medical University from January 2008 to April 2019. This study was approved by the Ethics Committee of the Children’s Hospital affiliated with Chongqing Medical University. Informed consent was obtained from the subjects and their legal guardians via signed consent forms.

EBV neurological infection diagnosis was confirmed by the positive antibodies of EBV capsule antigen IgM in the cerebrospinal fluid (CSF). EBV encephalitis/meningitis: The diagnostic criteria of viral meningitis were based on guidelines published in a 2010 issue of the European Journal of Neurology [6]. Acute myelitis, EBV-hemophagocytic lymphohistiocytosis (EBV-HLH), NS-post-transplant lymphoproliferative disorder (NS-PTLD), acute disseminated encephalomyelitis (ADEM), and Guillain–Barré Syndrome (GBS) were diagnosed based on clinical manifestations, laboratory examinations, and the clinical diagnostic criteria [7–11].

We used the anti-EBV capsule antigen antibody IgM kit (Euroimmun Medical Laboratory Diagnostics Stock Company, Zhejiang China) to detect EBV antibodies, and detected blood and CSF samples by enzyme-linked immunosorbent assay (ELISA). EBV-PCR detection was carried out using an EBV nucleic acid quantitative kit (Sansure Biotech, Hunan, China), and the PCR-fluorescence probe method was used to detect blood and CSF samples.

### Statistical methods

The results were analyzed by SPSS 21 statistical software. Normally distributed data were expressed as^−^means±standard deviation, and non-normally distributed data were expressed as medians (interquartile range). Numerical data were expressed as the number of cases and percentage (%), categorical variables were compared by chi-square test, and intergroup numerical variables were compared by independent samples *t*-test. *P* < 0.05 indicated statistical significance.

## Results

### Patient demographics and seasonal infection

**Fig. 1:**
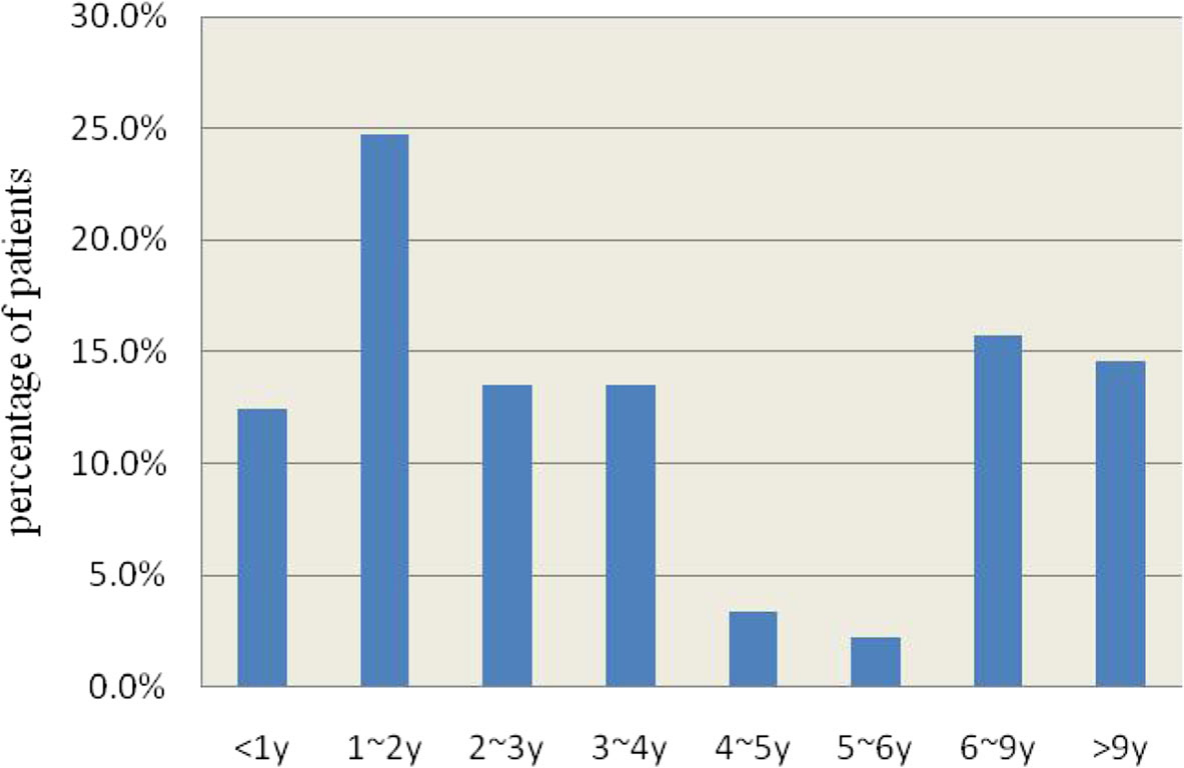
Age distribution of EBV cases involving the CNS

**Fig. 2:**
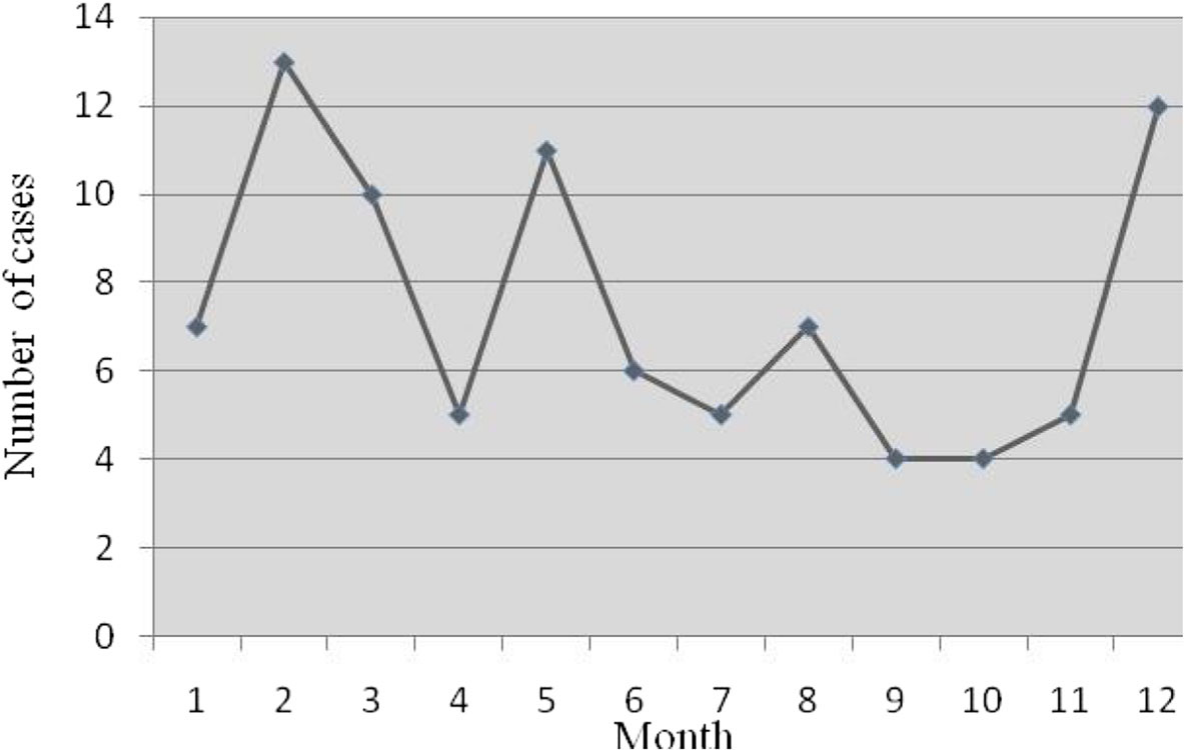
Monthly distribution of EBV cases involving the CNS

The study included 46 male and 43 female patients, with a male-to-female ratio of 1.07:1. Patient demographics are summarized in Fig. 1. The median age was 3 years (range: 1–191 months). The frequency of EBV infection with neurologic complications significantly varied between age groups (χ^2^ = 28.854, *P* < 0.001), peaking at 0–4 years (57/89, 64%). The EBV infection rate seemed well distributed across all seasons, but the months from December to February of the second year (winter) accounted for 36% of the total cases (spring: from March to May, 29.2%; summer: from June to August, 20.2%; autumn: from September to November, 14.6%), indicating a significant seasonality to the epidemic (*P* < 0.001, χ^2^ = 12.749, Fig. 2).

### Fever and fever duration

The definition of fever was an axillary temperature > 37.5 °C. Among the 89 cases, 74 cases (83.1%) had different degrees of fever. The mean fever duration was 9.31 ± 9.24 days (median: 8 days), and the thermal spike was 39.41 ± 0.84 °C (median: 39.5 °C). Details of the thermal spike and thermal duration are shown in Tables 1 and 2.

**Table 1:** Fever characteristics of pediatric patients with nervous system EBV infection

Temperature	*n*	Constituent ratio (%)
Normal body temperature	15	16.9
37.6–38 °C	10	11.2
38.1–39 °C	15	16.9
39.1–40 °C	34	38.2
> 40 °C	15	16.9
total	89	100

**Table 2:** The duration of fever in children with neurological impairment caused by EBV infection

Days	*n*	Percentage
≤ 3d	20	27.0
4-7d	16	21.6
8 − 14d	24	32.5
>14d	14	14.9
total	74	100

### Clinical manifestations of specific neurologic complications

The frequency of neurologic complications caused by EBV varied significantly (χ^2^ = 245.191, *P* < 0.001). The viral encephalitis/meningoencephalitis (71.9%) was the most common, followed by GBS (16.9%), neurologic damage caused by EBV-HLH (4.5%), ADEM (3.4%), acute myelitis (2.2%), and NS-PTLD (1.1%). (Table 3).

**Table 3:** Major clinical manifestations of nervous system damage caused by EBV

Symptoms (Number of cases)	Encephalitis/meningoencephalitis (*n* = 64)	Myelitis (*n* = 2)	EBV-HLH (*n* = 4)	NS-PTLD (*n* = 1)	ADEM (*n* = 3)	GBS (*n* = 15)
Fever	60	2	4	1	3	4
Intracranial hypertension	37	0	1	0	1	2
Drowsy-lethargy	7	0	1	0	1	2
Coma	15	0	0	0	2	0
Convulsions (not including SE)	21	0	0	0	0	0
Status epilepticus	8	0	0	1	2	0
Mental symptoms	4	0	0	0	0	0
Central respiratory failure	8	0	1	0	1	0
Peripheral respiratory failure	1	1	0	0	0	3
Ataxia	3	0	0	1	1	0
Facial nerve	5	0	0	0	2	1
Oculomotor nerve	0	0	0	0	1	2
Bulbar paralysis	0	0	0	0	0	10
Pyramidal tract sign	24	2	0	0	1	0
Meningeal irritation sign	24	1	0	0	3	9
Increased muscle tension	13	1	0	0	2	0
Decreased muscle tension	7	2	0	0	2	9
Decreased muscle strength	9	2	0	0	2	15
Defecation disorder	0	1	0	0	0	3
Urination disorder	1	0	0	0	1	4
Nerve root pain	0	1	0	0	0	2

*SE* Status epilepticus, *EBV-HLH* EBV-hemophagocytic lymphohistiocytosis, *NS-PTLD* NS-post-transplant lymphoproliferative disorder, *ADEM* acute disseminated encephalomyelitis, *GBS* Guillain–Barré Syndrome

One viral encephalitis patient developed recurrent convulsions and progressive disturbance of consciousness during the recovery period, secondary anti-NMDAR encephalitis was diagnosed 6 weeks after disease onset.

In two patients with acute myelitis, one had root pain that manifested as limb and back pain, while the other patient had diaphragm paralysis and constipation. Neither patient had encephalopathy or paresthesia.

One patient with ADEM and further complicated with peripheral nerve involvement was diagnosed as having acute disseminated encephalomyelitis and polyneuropathies (ADEMP).

### Laboratory test results

In all, 32 patients (36.0%) showed high peripheral blood leukocytes (range: 10.27–44.41 × 10^9^/L). Lymphocytes were dominant in the peripheral blood of 26 patients (29.2%).

Twenty-one children (23.6%) had abnormal liver function. Cardiac markers and myocardial enzymes showed abnormal results in 16 patients (16/82, 14.3%).

All included patients underwent complete CSF and blood examination. All the patients had positive antibodies of EBV capsule antigen IgM in the CSF. Further, 51 children (51/89, 57.3%) patients had positive antibodies of EBV capsule antigen IgM in the blood. Cerebrospinal fluid examination of EBV encephalitis/meningitis showed abnormal results in 46 cases (46/64, 71.9%); in 19 patients (19/64, 29.7%), the WBC count was mildly raised, 7 patients (7/64, 10.9%) had leukocyte count of > 100 × 10^6^/L, 16 patients (16/23, 69.6%) had mainly monocytes. The protein content was slightly increased in 24 patients (24/64, 37.5%); it was > 1 g/L in 14 patients (14/64, 21.9%). Two children with myelitis had a slight increase in the number of cells and proteins concentration, and monocytes were predominantly found in their WBC count. Among those with EBV-HLH, one patient had no CSF abnormality, one had increased leukocyte count in the CSF (mostly monocytes), one had slightly increased proteins, and one showed a significant increase in both protein content and number of nucleated cells. The leukocyte count increased slightly in children with NS-PTLD, and monocytes accounted for most of the leukocytes. The increase of CSF protein was noted in three cases of ADEM, and a slight increase of CSF cells (mainly monocytes) was only seen in two cases. The CSF protein level was increased in all 15 patients with GBS; 13 of these 15 patients (86.7%) had > 1 g/L protein, 14 (93.3%) had normal CSF cells, and one patient showed a slight increase in the CSF cell number (6.7%, 56 × 10^6^/L). Levels of CSF glucose and chloride were normal in all children. Six patients underwent CSF EBV-PCR examination, and 11 underwent blood EBV-PCR examination. Cerebrospinal fluid EBV-PCR was positive in three cases (50%), and blood EBV-PCR was positive in five (45.54%). The EBV loads in CSF and blood are presented in Table 4.

**Table 4:** The EBV loads in CSF and blood

	diagnosis	CSF load	CSF test time (days)	Blood load	Blood test time (days)
case1	EBV-HLH	1.07*10^4^	21	1.55*10^3^	13
		3.01*10^5^	23	3.98*10^5^	22
		7.77*10^4^	32	N*	29
case2	encephalitis	7.45*10^4^	24	2.52*10^3^	23
case3	ADEM	N	10	N	10
case4	encephalitis	-*	–	N	3
case5	encephalitis	–	–	N	3
case6	encephalitis	N	15	–	–
case7	NS-PTLD	–	–	N	-1*
				N	6
				N	11
				N	17
				8.67*10^5^	34
				2.18*10^7^	41
				1.64*10^8^	47
				5.55*10^9^	60
				1.49*10^4^	70
case8	EBV-HLH	–	–	6.3*10^5^	7
				1.07*10^7^	75
case9	encephalitis	N	10	N	10
case10	encephalitis	–	–	N	5
case11	encephalitis	–	–	N	7
case12	encephalitis	1.13*103	7	9.37*104	8

N: indicates the test outcome is negative

-: indicates incomplete test

− 1 day: means one day before allogeneic hematopoietic stem cell line transplantation, and the neurological system was affected 50 days after allogeneic hematopoietic stem cell line transplantation

In all, 53 encephalitis/meningitis patients were examined with brain MRI. The MRI results of 22 encephalitis/meningitis patients (41.5%) were abnormal, including 18 acute inflammatory edema, two cerebral atrophy, one encephalomalacia and cerebral atrophy, and one intracranial hemorrhage. The MRI results of three children with ADEM showed demyelination and blurred boundary of the lesion. Of these children, one showed involvement of white matter, deep nucleus, and brainstem, and another one had extensive brain lesions with signs of cerebral hernia.

Spinal cord MRI examination was performed in 12 children. The spinal cord and nerve root were involved in two patients with acute myelitis. In the patients with GBS, nerve roots were involved in five patients, spinal cord involvement was seen in one patient, and three patients showed normal MRI.

Peripheral nerve conduction: 17 patients were examined by peripheral nerve conduction, including 15 patients with GBS, one with acute myelitis, and another with ADEMP. All 17 cases showed decreased amplitude of peripheral motor nerve, and the conduction velocity decreased significantly in 6 of 17 cases (40%).

Electroencephalogram (EEG): EEG was performed in 64 patients, of whom 55 had encephalitis/meningitis. EEG was abnormal in 36 cases with encephalitis/meningitis: 29 cases showed background moderation and the remaining 7 showed epileptic discharge. Four cases of EBV-HLH showed background moderation. Three cases of ADEM showed significant background moderation, wherein one case was complicated with epileptic discharge. The EEG was normal in two children with GBS.

### Treatment and prognosis

After admission to the hospital, all the patients were given symptomatic support treatment, mainly for pyrexia, sedation, nerve nutrition, and to lower intracranial pressure. Eighteen patients with severe viral encephalitis were treated with IVIG, and 25 patients with EBV encephalitis and one with myelitis received antiviral therapy with acyclovir or ganciclovir.

All cases were followed up from 3 months to 139 months. The prognosis is shown in Table 5.

**Table 5:** Prognosis of different neurologic complications caused by EBV

	Encephalitis/meningitis (64 cases)	Acute myelitis (2 cases)	EBV-HLH (4 cases)	NS-PTLD (1 case)	ADEM (3 cases)	GBS (15 cases)
Lost to follow-up	7	1	1	0	0	0
Death	4	0	1	0	2	0
No sequelae	44	0	2	1	0	11
Mental retardation	1	0	0	0	0	0
Dyskinesia	1	0	0	0	0	0
Urination disorders	1	0	0	0	1	0
Total development retardation	5	0	0	0	0	0
Secondary epilepsy	2	0	0	0	0	0
Decreased muscle strength	0	1	0	0	0	0
Gait abnormality	0	0	0	0	1	3
Nerve root pain	0	0	0	0	0	1

## Discussion

The gold standard of encephalitis diagnosis is virus isolation in cell culture, but it has now been replaced by the detection of specific nucleic acid from the CSF or brain (Class Ia). Additionally, intrathecal antibody production to a specific virus is also strong evidence for etiology (Class Ib) [6]. However, virus detection from blood as well as systemic serological responses such as seroconversion or a specific IgM detection provides less strong evidence. Antibodies to EBV are measured from serum and CSF by enzyme immunoassay (EIA) tests. These tests are sensitive enough to detect even low amounts of CSF antibodies. Antibody levels in serum and CSF are compared at the same dilution of 1:200. If the ratio of antibody levels is < 20, it indicates intrathecal antibody production, provided that no other antibodies are present in the CSF [6]. The presence of specific IgM in the CSF indicates CNS disease. Besides, detection of specific nucleic acid from the CSF is dependent on the timing of the CSF sample, and PCR is associated with false-positive and false-negative results in EBV [12]. EBV-VCA-IgM in CSF was used as the diagnostic criteria of EBV neurological infection in our study. All included patients underwent complete examination of the CSF and blood. All patients had positive antibodies of EBV capsule antigen IgM in the CSF. Moreover, 57.3% (51/89) patients had positive antibodies of EBV capsule antigen IgM in the blood. Positive results of EBV-PCR were seen more in the middle and late stages than early stages.

EBV infection occurs worldwide, wherein about 90–95% of adults show positive titers for EBV serum antibodies. The seroepidemiological investigation of EBV infection in hospitalized children showed that the cumulative infection rate of EBV was nearly half of all preschool children, and the peak age of infection was 3–5 years. The infection rate was higher in March, September, and October. A serological study of 1364 children infected with EBV in Xinjiang showed that autumn and winter were the epidemic seasons [13]. In Shanxi, China, EBV infection rates show an increasing trend in autumn and winter as compared to spring and summer [14]. Our study showed that winter (December–February of the second year) was the epidemic season. This is likely related to the geographical differences between different regions, and we included children with neurologic damage caused by EBV infection as our study subjects. The disease can affect people of all ages, and the peak age among pediatric infection cases was 0–4 years, accounting for 64%, which is consistent with the epidemiological data from other studies.

There were significant clinical differences in neurological complications caused by EBV, including viral encephalitis/meningoencephalitis in 64 cases (71.9%), acute myelitis in two cases (2.2%), ADEM in three cases (3.4%), and GBS in 15 cases (16.9%). Neurologic damage caused by EBV-HLH was observed in four cases (4.5%) and NS-PTLD in 1 case (1.1%).

1. Viral encephalitis/meningitis: in our study, viral encephalitis/meningoencephalitis accounted for 71.9% (64/89), which was the most common neurological complication caused by EBV infection. A study about viral encephalitis in northern China showed that among the meningitis-encephalitis spectrum with definite etiology, the proportion of EBV infection is 5.8–6.6% [15]. In Hainan of China, a study about etiological analysis of viral encephalitis showed that the proportion of encephalitis caused by EBV was 6.5% (6/92) [16]. A clinical study in the University of Toronto, Canada, showed that 9.7% (21/216) of children with viral encephalitis were serologically positive and/or PCR positive for EBV [17]. In the etiological analysis of encephalitis reported by Alexandra Maille in France in 2007, encephalitis caused by EBV was about 2% (3/131) [18]. A study by Hamad Medical Center in Qatar show that EBV encephalitis was as high as 31% (65/218) in viral encephalitis due to identified pathogens [19]. Therefore, EBV should be included as a routine etiological test for suspected NS infection.

Our study found that the clinical manifestations of EBV encephalitis were not specific. The main manifestations were acute onset fever seen in 93.8% cases (60/64). The symptoms of intracranial hypertension such as headache and vomiting were seen in 37/64 patients (57.8%): some of them were accompanied with different degrees of consciousness disturbance (22/64, 34.4%); convulsions (29/64, 45.3%); and even status epilepticus (8/64, 12.5%), similar to the results reported by Doja [17]. Some patients showed ataxia (3/64, 4.7%) or were complicated with cranial nerve involvement (5/64, 7.8%). Central respiratory failure can occur when the brainstem is involved. Cranial nerve involvement could be the first symptom of EBV encephalitis [20].

In this study, the children with EBV-related encephalitis/meningitis had no symptoms of EBV infection outside the NS, such as tonsillitis, lymph node enlargement, skin rash, and hepatosplenomegaly. It is suggested that EBV encephalitis in children may be a primary infection of the NS, which supports the notion that neurological damage is caused by direct invasion of EBV. However, pediatric infectious mononucleosis may be considered less severe if they have only mild neurological symptoms such as simple mental fatigue and self-limited encephalitis.

One patient developed anti-NMDAR encephalitis during the recovery period of viral encephalitis. Anti-NMDAR encephalitis may be related to infection. The pathogens reported at present include herpes simplex virus, influenza virus, *Mycoplasma pneumoniae*, human herpes zoster virus, enterovirus, measles virus, and Japanese encephalitis virus. Among them, herpes simplex virus has been considered to be most closely related to anti-NMDAR encephalitis in recent years. It is speculated that the appearance of anti-NMDAR antibody after virus infection may be the result of brain infiltration of inflammatory, injured, and necrotic neuronal cells’ exposed surface antigen, a break in the immune tolerance, and subsequent production of corresponding antibodies. Other receptor proteins on the surface of neurons may also be involved. Similar to the role of *Enterobacter jejuni* infection in GBS, viral infection may cause the body to directly produce antibodies to synaptic proteins through the mechanism of viral molecular mimicry or exposure to common antigen. Therefore, patients with delayed or recurrent encephalitis should be screened for anti-NMDAR and/or other synaptic protein antibodies to make a timely diagnosis, adjust the treatment plan, and improve prognosis.

The MRI results of 22 encephalitis/meningitis patients (22/53,41.5%) were abnormal, with the main manifestation being cytotoxic edema accounting for 33.9% (18/53). The other imaging findings included demyelination, cerebral atrophy, encephalomalacia, and hemorrhage. A study about the location of imaging and prognosis in Lund University, Sweden, showed that patients with focal gray matter or white matter involvement have a good prognosis, half the patients with thalamic involvement have sequelae, and patients with brainstem involvement have a high mortality rate. Abul-Kasim suggested that the neuroanatomic distribution of the radiological abnormalities in EBV encephalitis may be useful as a prognostic marker [21]. Brain MRI has important clinical significance. The EEG results were abnormal in 65.5% (36/55) of encephalitis/meningitis cases, including 29 cases with background moderation and seven with epileptic discharge. EEG changes in viral encephalitis are usually nonspecific, and background changes can occur before imaging abnormalities can be detected. The detection rate of abnormal CSF was 71.9% (46/64), which was similar to 84.2% [22] reported in the literature. The CSF of encephalitis/meningitis caused by EBV showed non-specific changes. Cerebrospinal fluid routing showed a slight increase in leukocytes, generally < 100 × 10^6^ L, the majority of which were lymphocytes. The normal or slightly increase in CSF protein levels may be because of the increase of blood-brain barrier permeability that allowed plasma proteins into the CSF or an increase in the intrathecal inflammatory factors or structural proteins. The content of glucose in the CSF was normal or increased.

In our study, the overall prognosis of EBV encephalitis/meningitis was good, as 68.7% (44/64) patients recovered completely, and 14% (9/64) were left with varying degrees of sequelae including mental retardation, motor disorders, language disorders, defecation disorders, and secondary epilepsy. The main cause of death was respiratory and circulatory failure caused by brainstem involvement.

2. Guillain–Barré Syndrome: EBV infection can also lead to GBS, mainly caused by an abnormal immune cross response, resulting in peripheral nerve axonal injury and demyelination [23]. In our study, four children had fever in the course of disease, and 80.0% (12/15) of the children showed further complications with multiple cranial nerve damage, mainly damaged glossopharyngeal nerve, vagus nerve, and facial nerve, which was consistent with a previous report [24]. GBS can be complicated with autonomic nerve damage such as hyperactivity of hands and feet, tachycardia or bradycardia, changes in blood pressure, and defecation disorders [25]. One patient had transient urinary retention in our study. Peripheral nerve conduction suggests that the main abnormality is peripheral nerve axonal neuropathy, about half of which is associated with abnormal myelin, considered as acute motor axonal neuropathy, also the main type of GBS in China, Japan, and other Asian countries [26]. Three patients (3/15, 20%) showed slight gait abnormality.

3.Others: Acute myelitis, ADEM, neurological damage caused by EBV-HLH, and EBV-related NS-post-transplant lymphoproliferative disorder case numbers were small. The main spinal cord injury caused by EBV infection was incomplete spinal cord injury [7]. The thoracic spinal cord was the most common segment of the spinal cord involved in EBV infection [27]. In this study, one case showed involvement of cervicothoracic spinal cord and the other, of the whole spinal cord. EBV infection of the NS can cause demyelination of the central NS or peripheral NS, or both simultaneously [28]. Molecular mimicry is recognized as a mechanism of NS demyelination induced by EBV. The peripheral NS myelin antigen P2 protein in GBS are attacked, and the myelin basic protein of the NS is attacked in ADEM. However, the attack of myelin antigen P1 protein in peripheral NS of ADEMP can cause demyelination of both the central and peripheral nerves [29]. Some researchers have speculated that GBS, Miller–Fisher syndrome, and ADEM are all acute immune neuropathies. The clinical manifestations of the children in this group were complex and diverse, and the degree of inflammation and prognosis were different, which were related to the location and severity of inflammatory demyelination [30]. A recent study showed no consensus on the definition of HLH-related NS diseases such as NS-HLH. Most experts reported that NS-HLH is activated lymphocytes and macrophages infiltrating the meninges and brain tissue; the CSF and/or brain MRI is abnormal, with or without obvious neurological signs/symptoms [31, 32]. In the study by Anna Carin Horne, 63% patients with HLH may have had neurological symptoms and/or abnormal CSF (122/193), including meningoencephalitis and severe neurological sequelae [33]. In the study of 89 children with HLH, 39 patients showed NS involvement [34]. The incidence of EBV-associated PTLD was about 5–15% [35]. The main manifestations were dizziness, headache, epilepsy, disturbance of consciousness, fever, fatigue, weight loss, and other systemic symptoms [36]. Most early-onset PTLD (occurred within 1 year after transplantation) was associated with recent EBV infection, and the correlation between late-onset PTLD and EBV infection was unremarkable [37]. The neurological involvement of PTLD patients in this study was seen 50 days after hematopoietic stem cell transplantation.

In our study, 25 patients with EBV encephalitis and one with myelitis received antiviral therapy. There are no guidelines for the treatment of EBV infection in the NS. The main treatment includes antiviral and symptomatic support therapy. Acyclovir and ganciclovir can effectively inhibit EBV replication, but the clinical therapeutic effect is limited. Ganciclovir is good at penetrating the blood-brain barrier, and the concentration in the brain tissue can reach 60% of the blood concentration, thus making it more effective than acyclovir in the treatment of EBV infection in the NS [38, 39]. According to the guidelines of the American Society of Infectious Diseases, intravenous acyclovir is not recommended for EBV-associated encephalitis [40]. At present, the main clinical application is using ganciclovir. However, liver function should be monitored when using antiviral drugs [41].

## Conclusions

There were significant differences in neurological complications caused by EBV. The prognosis of EBV infection in the NS is generally good. These illnesses are often self-limiting. A few cases may show residual sequelae.

## Data Availability

**A**ll data generated or analyzed during this study are included in this published article.

## Abbreviations

EBV: Epstein–Barr virus
NS: Nervous system
CSF: Cerebrospinal fluid
ADEM: Acute disseminated encephalomyelitis
GBS: Guillain–Barré Syndrome
EBV-HLH: EBV-hemophagocytic lymphohistiocytosis
NS-PTLD: NS-post-transplant lymphoproliferative disorder
EEG: Electroencephalogram
